# Development of novel SUV39H2 inhibitors that exhibit growth suppressive effects in mouse xenograft models and regulate the phosphorylation of H2AX

**DOI:** 10.18632/oncotarget.25806

**Published:** 2018-08-07

**Authors:** Theodore Vougiouklakis, Vassiliki Saloura, Jae-Hyun Park, Naofumi Takamatsu, Takashi Miyamoto, Yusuke Nakamura, Yo Matsuo

**Affiliations:** ^1^ Section of Hematology/Oncology, Department of Medicine, The University of Chicago, Chicago, IL, USA; ^2^ OncoTherapy Science Inc., Kawasaki, Japan; ^3^ Department of Surgery, The University of Chicago, Chicago, IL, USA

**Keywords:** methyltransferase, small-molecule inhibitor, SUV39H2, γ-H2AX

## Abstract

Protein methyltransferase SUV39H2 was reported to methylate histone H2AX at lysine 134 and enhance the formation of phosphorylated H2AX (γ-H2AX), which causes chemoresistance of cancer cells. We found that a series of imidazo[1,2-*a*]pyridine compounds that we synthesized could inhibit SUV39H2 methyltransferase activity. One of the potent compounds, OTS193320, was further analyzed in *in vitro* studies. The compound decreased global histone H3 lysine 9 tri-methylation levels in breast cancer cells and triggered apoptotic cell death. Combination of OTS193320 with doxorubicin (DOX) resulted in reduction of γ-H2AX levels as well as cancer cell viability compared to a single agent OTS193320 or DOX. Further optimization of inhibitors and their *in vivo* analysis identified a compound, OTS186935, which revealed significant inhibition of tumor growth in mouse xenograft models using MDA-MB-231 breast cancer cells and A549 lung cancer cells without any detectable toxicity. Our results suggest that the SUV39H2 inhibitors sensitize cancer cells to DOX by reduction of γ-H2AX levels in cancer cells, and collectively demonstrate that SUV39H2 inhibition warrants further investigation as a novel anti-cancer therapy.

## INTRODUCTION

A large body of evidence has demonstrated that deregulation of protein methyltransferases plays key roles in human tumorigenesis, which is mediated not only by chromatin modifications but also through direct non-histone protein methylation. As such, promising efforts have been made to target these enzymes. A DOT1L-specific methyltransferase inhibitor, EPZ004777, was shown to extend survival in a mixed lineage leukemia (MLL)-rearranged leukemia mouse model and attenuated global H3K79 methylation in human leukemia cell lines [1]. Subsequent studies with an improved DOT1L inhibitor, EPZ-5676, have exhibited complete regression of *MLL*-rearranged leukemia in a rat xenograft model [2]. Furthermore, an inhibitor of EZH2 methyltransferase activity, GSK126, resulted in reduction of H3K27 methylation levels *in vitro* and *in vivo*, and inhibited growth of EZH2-mutated diffuse large B-cell lymphoma (DLBCL) mouse xenografts and proliferation of EZH2-mutated DLBCL cell lines [3]. These lines of evidence delineate the encouraging nature of methyltransferase inhibition for the development of small-molecular inhibitors for cancer treatment.

DNA repair pathways play a fundamental role in maintaining the stability of the human genetic code and ensuring cell vitality. A hallmark of DNA damage is the formation of double strand breaks, which rapidly result in the formation of phosphorylated H2AX (Ser 139), also known as γ-H2AX[4–6]. These sites serve as a landing platform for the recruitment of repair proteins and other factors involved in DNA damage signaling [7, 8]. In cancer cells, DNA repair pathways promote survival and evasion of death following DNA-damaging chemotherapy or radiotherapy. Thus pharmacologic inhibition of DNA repair pathways has been investigated to reverse resistance to DNA-damaging chemotherapy. Efforts to inhibit pathways involved in DNA repair and augment the efficacy of DNA damaging anti-cancer agents have been investigated in phase I-II clinical trials [9–11].

SUV39H2 (Suppressor of variegation 3-9 homolog 2), also known as KMT1B (Lysine N-methyltransferase 1B), is a protein methyltransferase known to methylate histone H3 at lysine 9 (H3K9), resulting in heterochromatin formation and transcriptional repression. In recent years, our group has explored the biological importance of SUV39H2 to better understand the role of this methyltransferase in tumorigenesis [12–15]. We previously found that SUV39H2 methylates histone H2AX at lysine 134, which enhances the accumulation of γ-H2AX and enhances DNA repair activity in human cancer [12]. This methylation plays a crucial role in increasing chemo- and radio-resistance in cancer cells through the enhancement of γ-H2AX production by SUV39H2-dependent methylation. A search in The Cancer Genome Atlas (TCGA) database revealed SUV39H2 alterations (mutations, deletions, and amplifications) in a wide variety of human cancers, implying that alterations in this gene may play an important role in cancer development and progression. Furthermore, our expression profile analysis revealed SUV39H2 to be highly expressed in many cancer types, while its expression levels are very low or hardly detectable in normal tissues except adult testis [12]. Importantly, studies have indicated increased γ-H2AX expression levels in triple negative breast cancer (TNBC) and p53*-*mutated breast cancer cell lines [16]. Moreover, TNBCs with high γ-H2AX levels were found to portend a dismal prognosis [16]. Overexpression of γ-H2AX has also been shown to confer worse overall survival in patients with non-small cell lung cancer (NSCLC) and endometrial cancer [17, 18]. These findings imply that SUV39H2 has a critical role in human tumorigenesis and make it an attractive molecular target for anti-cancer therapeutics due to its selective expression in cancer cells. Henceforward, owing to the intricate relationship between SUV39H2 and γ-H2AX formation, we speculated that SUV39H2 inhibition may be a promising potential drug target in various types of human cancer.

In the present study, we report a series of imidazo[1,2-*a*]pyridine compounds that could inhibit SUV39H2 methyltransferase activity. We have characterized one of the newly synthesized compounds, OTS193320 [19], in detail *in vitro* and found that the treatment with OTS193320 significantly reduced global H3K9 tri-methylation (H3K9me3) in breast cancer cells *in vitro* and induced apoptotic cell death. This compound, OTS193320, attenuated γ-H2AX levels when used in combination with doxorubicin (DOX) compared with the DOX treatment alone, and caused a significant reduction in breast cancer cell viability when compared to the treatment with a single agent, OTS193320 or DOX. A further optimized compound, OTS186935 [19], revealed a significant growth inhibitory effect in mouse xenograft models using MDA-MB-231 breast cancer cells as well as A549 lung cancer cells. These results demonstrate that SUV39H2 inhibition may provide a novel therapeutic approach for treatment of various types of human cancer.

## RESULTS

### SUV39H2 knockdown significantly decreases the viability and levels of H3K9 tri-methylation in breast cancer cells

**Figure 1:**
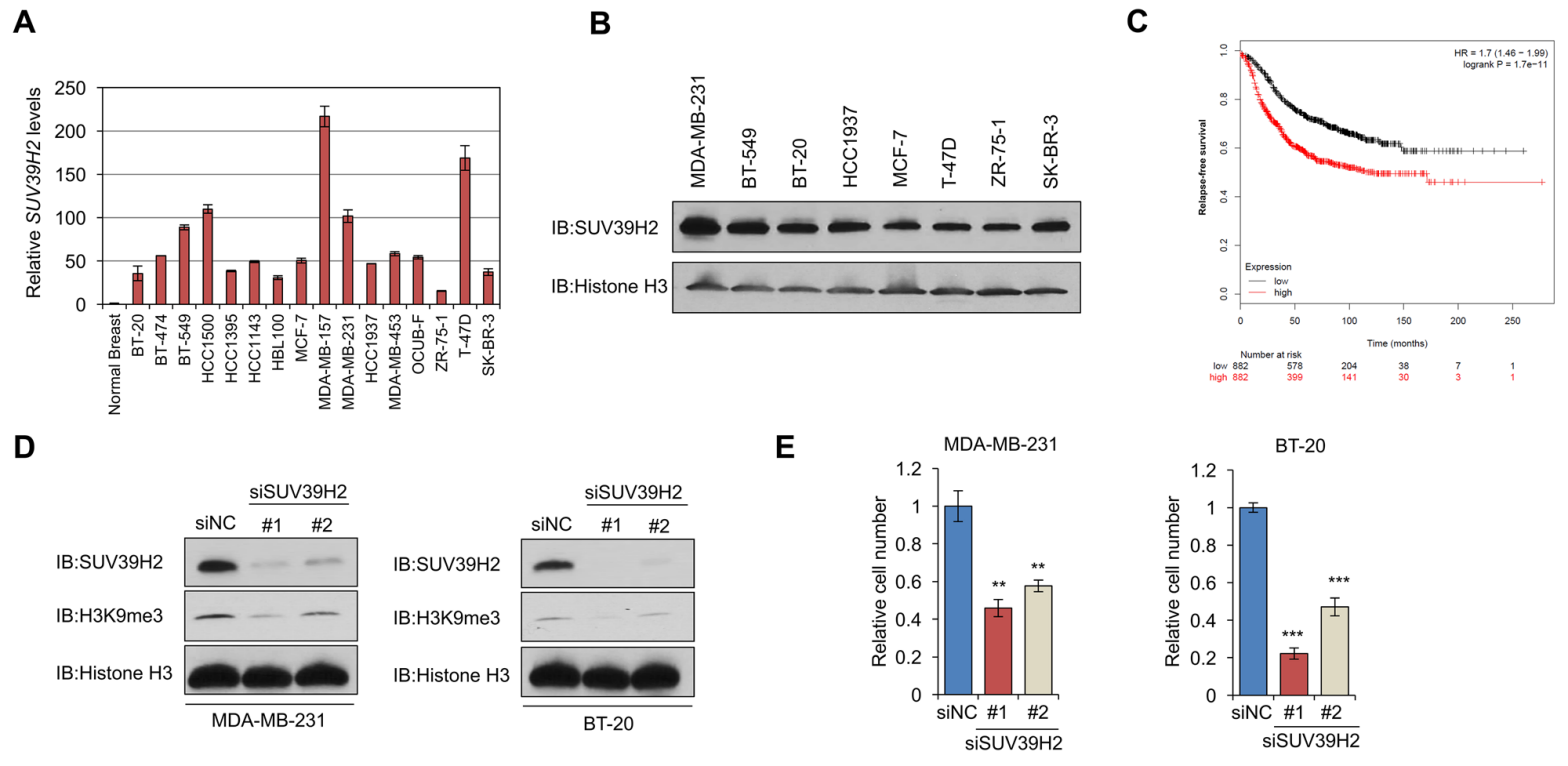
SUV39H2 is overexpressed in breast cancer cells and induces H3K9 tri-methylation **(A-B)** SUV39H2 overexpression in breast cancer cell lines. Expression levels were analyzed by quantitative real-time PCR (A) and by western blot (B). **(C)** Kaplan–Meier analysis of *SUV39H2* expression with relapse-free survival (RFS) of breast cancer patients (*P* = 1.7 x10^-11^). Data was obtained using the KM plotter; www.kmplot.com. Expression range of the *SUV39H2* probe (1554572_a_at) was 28 to 4984. The cutoff level used in the analysis was 324 to divide *SUV39H2*-high (*n* = 882) and *SUV39H2*-low (*n* = 882) groups. **(D)** Effect of *SUV39H2* knockdown on H3K9me3 levels. SUV39H2 knockdown attenuated global levels of H3K9me3 in cells transfected with siSUV39H2#1 or siSUV39H2#2, compared to control siRNA (siNC). **(E)** MTT assays of MDA-MB-231 and BT-20 cells. SUV39H2 knockdown causes significant growth suppression in cells treated with siSUV39H2. MTT assays were performed using the Cell Counting Kit-8. Relative cell numbers are normalized to the number of siNC-treated cells (siNC = 1). *P* values were calculated using Student’s *t* test (^**^P < 0.01; ^***^P < 0.001).

To determine whether SUV39H2 may possess important roles in human breast cancer, we examined the expression levels of *SUV39H2* in breast cancer cell lines using quantitative real-time PCR and identified that *SUV39H2* was significantly upregulated in all of the breast cancer cell lines, compared with a normal breast tissue (Figure 1A). Western blot analysis further confirmed high SUV39H2 expression levels at the protein level in a panel of cancer cell lines representative of different subtypes of breast cancer (Figure 1B). Since we observed *SUV39H2* overexpression in breast cancer cell lines, we examined the correlation between *SUV39H2* expression levels and relapse-free survival (RFS) in breast cancer patients across all subtypes from a published microarray [20] and found that high expression of *SUV39H2* was strongly correlated with poor prognosis (*P* = 1.7×10^-11^) using Kaplan–Meier analysis (Figure 1C).

We subsequently examined the effect of SUV39H2 knockdown using small interfering RNAs (siRNAs) on the methylation status of H3K9, a known substrate of SUV39H2. We transiently transfected either of two SUV39H2-specific siRNAs (siSUV39H2#1 and siSUV39H2#2) into two TNBC cell lines, MDA-MB-231 and BT-20, in which SUV39H2 was overexpressed and prepared nuclear extracts from harvested cells. Western blot analysis demonstrated significant downregulation of SUV39H2 at the protein level and attenuation of H3K9me3 levels in the siSUV39H2-treated cells compared to those treated with a control siRNA (siNC) (Figure 1D). To further examine the importance of SUV39H2 in cancer cell viability, we conducted MTT assays using MDA-MB-231 and BT-20 cells. Knockdown of *SUV39H2* in these two cell lines significantly decreased cell viability, compared to those treated with siNC (Figure 1E), suggesting that SUV39H2 plays a critical role in breast cancer cell growth.

### Identification of a novel SUV39H2 inhibitor

**Figure 2:**
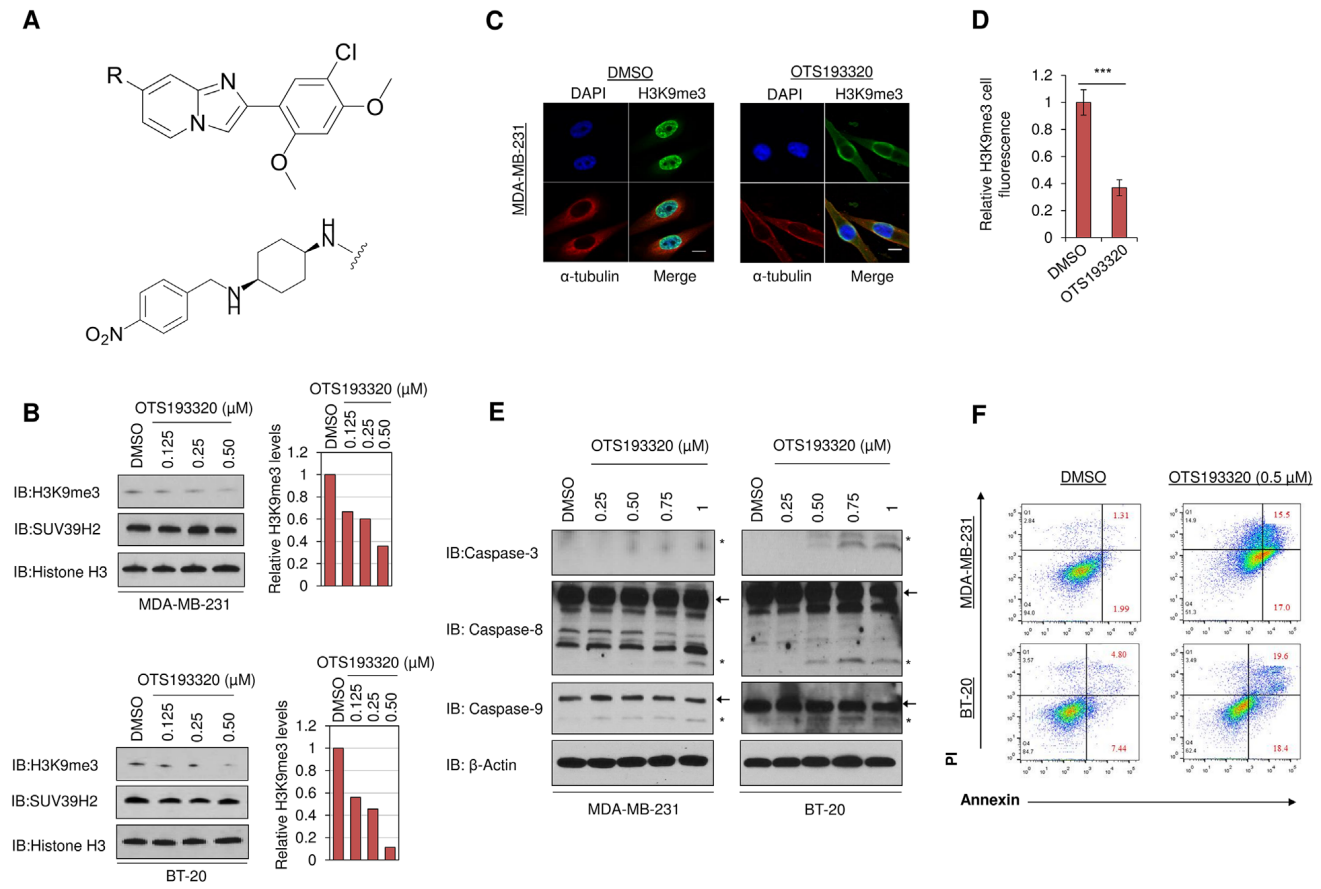
Chemical structure of the novel SUV39H2 inhibitor and *in vitro* characterization of OTS193320 The novel SUV39H2 methyltransferase inhibitory compound, OTS193320 (**A** lower panel) shares a common imidazo[1,2-*a*]pyridine derivative structure (shown in **A** top panel). The compounds with different substituent groups R at the 7-position of the imidazopyridine scaffold, including OTS193320 with ((1*s*,4*s*)-*N*^1^-(2-(5-chloro-2,4-dimethoxyphenyl)imidazo[1,2-*a*]pyridin-7-yl)-*N*^4^-(4-nitrobenzyl)cyclohexane-1,4-diamine), were synthesized. The R-groups of OTS193320 are shown in (A lower panel). **(B)** Dose-dependent inhibition of H3K9me3 in MDA-MB-231 and BT-20 cells after exposure to OTS193320 for 24 hours. X-ray films were scanned with GS-800^™^ calibrated densitometer (Bio-Rad) and bar charts depict the relative decrease of H3K9me3 compared to DMSO. **(C)** Immunocytochemical analysis demonstrating attenuation of H3K9me3 in MDA-MB-231 cells after exposure to 0.5μM of OTS193320 for 24 hours. Cells were stained with an anti-α-tubulin antibody (red), an anti-H3K9me3 antibody (green) and 4’,6’-diamidine-2’-phenylindole dihydrochloride (DAPI, blue). Scale bars are 10 μm. **(D)** Nuclear H3K9me3 intensity was quantified using the Image J software and results are the mean ± s.d. of ten independent cells. *P* values were calculated using Student’s *t* test (^***^P < 0.001). **(E)** Induction of apoptotic markers in MDA-MB-231 and BT-20 cells treated with different concentrations of OTS193320 for 48 hours. Western blot analysis demonstrated a dose-dependent increase of cleaved caspase-3, -8, and -9. Arrows denote full length fragments and asterisks represent the cleaved fragments. **(F)** Apoptosis assessed by Annexin and PI staining in MDA-MB-231 and BT-20 cells. Cells were treated with 0.5μM of OTS193320 or DMSO (control) for 48 hours. Numbers in red color represent the average percentage of early and late apoptotic cells.

Through screening of a commercially available compound panel, we identified some imidazo[1,2-*a*]pyridine derivative compounds to have weak SUV39H2 methyltransferase inhibitory activity with IC_50_ (half maximal inhibitory concentration) values of <10 μM. Through intensive structure-activity relationship-based drug development, we obtained novel compounds that shared a common chemical structure as shown in Figure 2A top panel. Among them, a compound, OTS193320 (Figure 2A lower panel), exhibited a high inhibitory effect against SUV39H2 enzymatic activity (IC_50_ of 22.2 nM) and a growth suppressive effect of SUV39H2-positive A549 lung cancer cells (IC_50_ of 0.38 μM).

We further examined the *in vitro* growth inhibitory effect of OTS193320 on MCF-7, SK-BR-3, ZR-75-1, T-47D, MDA-MB-231, and BT-20 breast cancer cell lines, and found IC_50_ values from 0.41 to 0.56 μM, respectively (Supplementary Figure 1). We then investigated the effect of this compound on H3K9me3 levels in breast cancer cells. Incubation of MDA-MB-231 and BT-20 cells with OTS193320 for 24 hours caused attenuation of H3K9me3 levels in a dose-dependent manner as shown in the western blot analysis (Figure 2B). To confirm the suppression of H3K9me3 in cancer cells, we performed immunocytochemical analysis of MDA-MB-231 cells after exposure to OTS193320. Immunocytochemical analysis showed a drastic reduction of the H3K9me3 methylation signal in cells treated with OTS193320, consistent with the western blot analysis (Figure 2C, 2D). These findings imply that SUV39H2 enzymatic methyltransferase activity is strongly suppressed by OTS193320.

### OTS193320 induces apoptosis in breast cancer cells

To examine whether inhibition of SUV39H2 methyltransferase activity by OTS193320 mediates breast cancer cell death, we treated MDA-MB-231 and BT-20 cells with OTS193320 for 48 hours and performed western blot analysis. We identified activation of caspases in both cell lines in an OTS193320 dose-dependent manner (Figure 2E). Furthermore, we stained cells with Annexin V and propidium iodide (PI) and performed flow cytometric analysis to quantitatively examine the percentage of apoptotic cells following treatment with the compound. Cells treated with OTS193320 for 48 hours showed an increase in the number of cells at early- and late-stage apoptosis, as demonstrated by the percentage of Annexin V and PI positive cells (Figure 2F). These data strongly support that SUV39H2 inhibition by OTS193320 induces apoptotic cell death of breast cancer cells.

### OTS193320 sensitizes breast cancer cells to doxorubicin via attenuation of γ-H2AX

**Figure 3:**
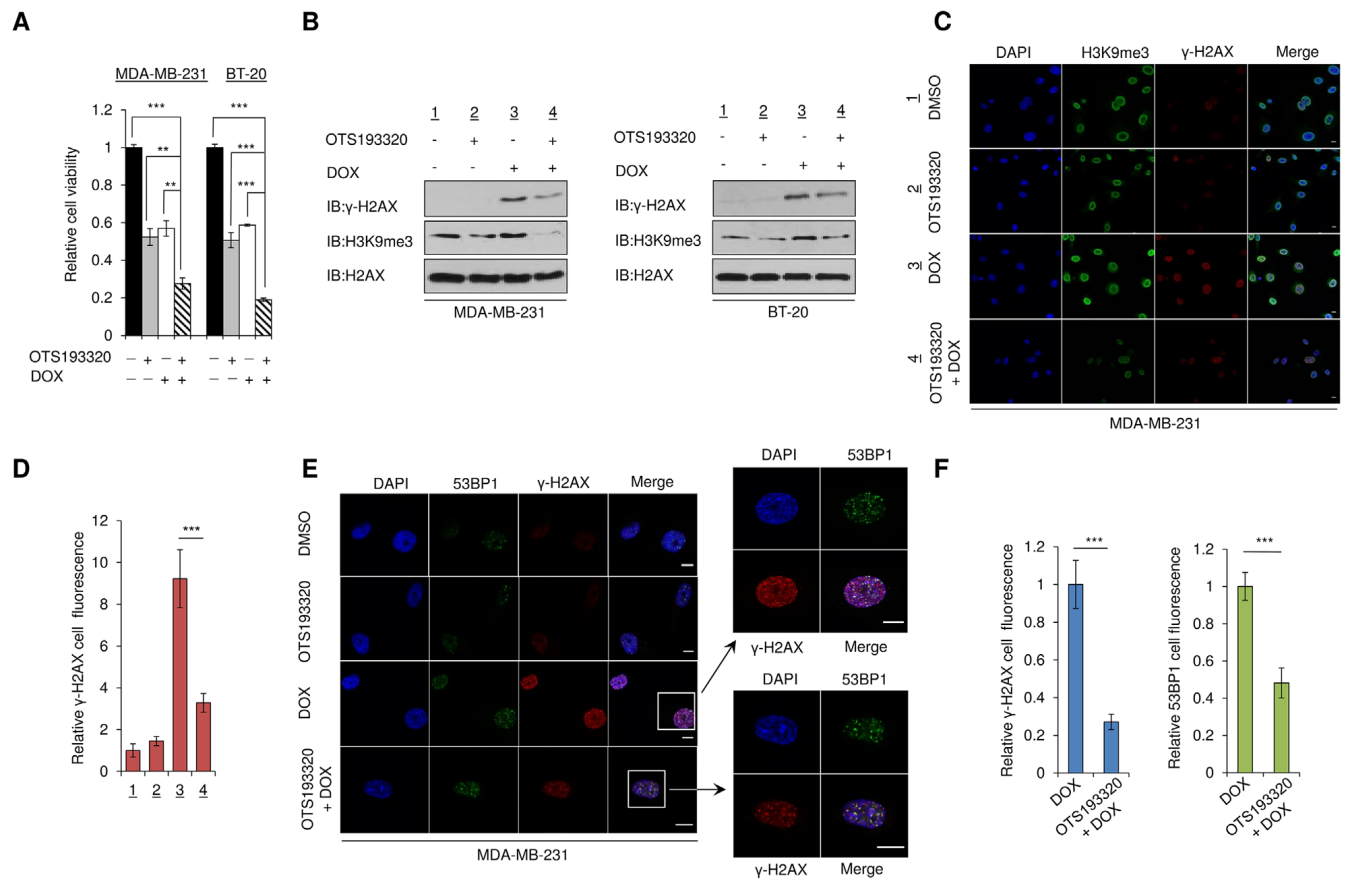
Combination of OTS193320 and DOX attenuates levels of γ-H2AX in cancer cells **(A)** MTT assays of MDA-MB-231 and BT-20 cells after exposure to the concentration of IC_50_ values of OTS193320, doxorubicin (DOX), or the combination. MTT assays were performed using the Cell Counting Kit-8. Relative cell numbers are normalized to the number of cells exposed to DMSO (DMSO = 1). *P* values were calculated using Student’s *t* test (^**^P < 0.01; ^***^P < 0.001). **(B)** MDA-MB-231 and BT-20 cells were incubated with the concentration of IC_50_ values of OTS193320, DOX, or the combination for 12 hours, followed by histone purification. Western blot analysis shows reduction of γ-H2AX in the combination treatment compared with DOX treatment alone. **(C)** Immunocytochemical analysis of MDA-MB-231 cells demonstrating reduction of γ-H2AX in the combination treatment compared with DOX treatment alone after incubation for 12 hours with the compounds. Cells were stained with an anti-γ-H2AX antibody (red), anti-H3K9me3 antibody (green) and 4’,6’-diamidine-2’-phenylindole dihydrochloride (DAPI, blue). Scale bars are 10 μm. **(D)** Nuclear γ-H2AX intensity was quantified with the Image J software, and results are the mean ± s.d. of ten independent cells. *P* values were calculated using Student’s *t* test (^***^P < 0.001) **(E)** Immunocytochemical analysis of MDA-MB-231 cells demonstrating reduction and co-localization of γ-H2AX and 53BP1 in the combination treatment compared with DOX treatment alone. Cells were incubated with the concentration of IC_50_ values for 12 hours. Cells were stained with an anti-γ-H2AX antibody (red), anti-53BP1 antibody (green) and 4’,6’-diamidine-2’-phenylindole dihydrochloride (DAPI, blue). Scale bars are 10 μm. **(F)** Nuclear γ-H2AX and 53BP1 intensities were quantified using the Image J software. Results are the mean ± s.d. of ten independent cells. *P* values were calculated using Student’s *t* test (^***^P < 0.001).

Previous work in our laboratory demonstrated that SUV39H2-mediated H2AX methylation increases the formation of γ-H2AX in lung cancer cells and that inhibition of SUV39H2 enhances chemosensitivity of lung cancer cells to cisplatin or DOX [12]. To examine whether OTS193320 can regulate the formation of γ-H2AX in MDA-MB-231 and BT-20 breast cancer cells, we investigated this compound as a single agent and in combination with DOX. MDA-MB-231 and BT-20 cells were exposed to OTS193320, DOX, or the combination of both compounds. Incubation of the two cell lines with both OTS193320 and DOX significantly attenuated cancer cell viability *in vitro*, compared to single agent treatment of either drug (Figure 3A). Subsequently, we performed western blot analysis using purified histone extracts from these two cell lines. Combination of OTS193320 and DOX reduced γ-H2AX expression levels compared to DOX treatment alone, indicating that OTS193320 suppressed γ-H2AX generation by DOX through inhibition of H2AX methylation, which is essential for the formation of γ-H2AX (Figure 3B). To further validate the reduction of γ-H2AX in the combination therapy, we performed immunocytochemical analysis using MDA-MB-231 cells. Concordant to the western blotting, we observed significant reduction in γ-H2AX in the combination therapy compared with DOX mono-treatment, further supporting that OTS193320 plays a critical role in the regulation of γ-H2AX formation in breast cancer cells (Figure 3C, 3D). Subsequently, we examined the effect of combination therapy on the localization of p53 binding protein 1 (53BP1) because 53BP1 is reported to co-localize with γ-H2AX foci at DNA damage sites and high 53BP1 foci confer poorer prognosis in TNBC patients [8, 16]. Expectedly, we observed co-localization, but attenuation of γ-H2AX and 53BP1 levels in the combination therapy (Figure 3E, 3F). Taken together, these results demonstrate that OTS193320 plays a crucial role in the regulation of γ-H2AX production in human breast cancer cells.

### OTS186935 exhibits growth suppressive effects in human cancer cell line derived xenograft models

**Figure 4:**
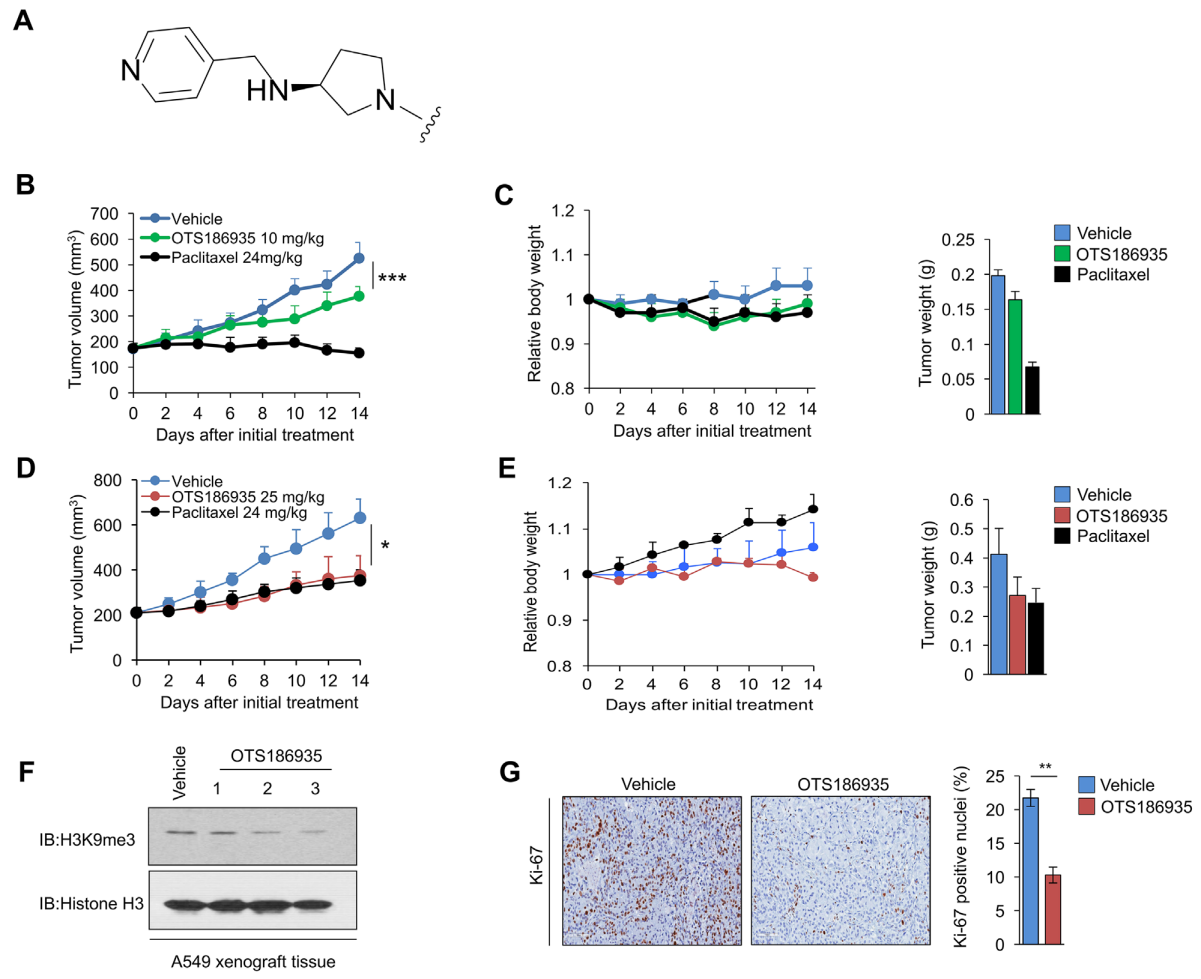
*In vivo* efficacy of OTS186935 in MDA-MB-231 and A549 xenograft mouse models **(A)** OTS186935 shares a common imidazo[1,2-*a*]pyridine derivative structure as shown in Figure 2A top panel. The compound OTS186935 ((*S*)-1-(2-(5-chloro-2,4-dimethoxyphenyl)imidazo[1,2-*a*]pyridin-7-yl)-*N*-(pyridin-4-ylmethyl)pyrrolidin-3-amine) was synthesized with a different substituent group R at the 7-position of the imidazopyridine scaffold. The R-group of OTS186935 is depicted in (A). **(B-C)** Mice bearing MDA-MB-231 breast cancer cells (*n* = 6 per group) were intravenously treated with OTS186935 or vehicle control once daily for 14 days. (B) Mean tumor volumes + s.d. are shown. Intravenous administration of OTS186935 at 10 mg/kg resulted in tumor growth inhibition (TGI) of 42.6%. *P* values were calculated using Student’s *t* test (^***^P < 0.001). (C) Mean relative body weights + s.d. compared to mean body weight prior to administration of compounds (day 0), and mean tumor weight + s.d. on day 14 are shown. **(D-E)** Mice bearing A549 lung cancer cells (*n* = 3 per group) were intravenously treated with OTS186935 or vehicle control once daily for 14 days. (D) Mean tumor volumes + s.d. are shown. Intravenous administration of OTS186935 at 25 mg/kg resulted in tumor growth inhibition (TGI) of 60.8%. *P* values were calculated using Student’s *t* test (^*^P < 0.05). (E) Mean relative body weights + s.d. compared to mean body weight prior to administration of compounds (day 0), and mean tumor weight + s.d. on day 14 are shown. **(F)** OTS186935 attenuated the levels of H3K9me3 in A549 xenograft mouse model. Western blot demonstrates H3K9me3 levels in OTS186935-treated mice (*n* = 3) compared to vehicle control. **(G)** Representative immunohistochemical staining examples of Ki-67 levels in vehicle control and OTS186935-treated mice in the A549 xenograft model (*n* = 3 per group). OTS186935 significantly attenuated number of Ki-67 positive nuclei in the mice treated with the compound compared to vehicle control. The percentage of positive nuclei and intensity scores were calculated from vehicle control and OTS186935-treated mice (*n* = 3 per group). *P* values were calculated using Student’s *t* test (^**^P < 0.01). Slides were scanned using ScanScope XT and the analysis was conducted using Aperio Nuclear Image Analysis algorithm in three different optical fields within the slide sections (Magnification of 20X).

We applied a mouse xenograft model using MDA-MB-231 cells to examine the *in vivo* efficacy of OTS193320, but no growth inhibitory effect was observed in dose ranges that revealed no toxic events. Hence, we further synthesized and screened additional compounds and found one compound, OTS186935 (Figure 4A), that showed enzymatic IC_50_ of 6.49 nM and A549 cell growth inhibitory effect with IC_50_ of 0.67 μM. We then examined the growth inhibitory effect of OTS186935 using MDA-MB-231 TNBC cells. The immunodeficient mice harboring MDA-MB-231 (*n* = 6) were administered OTS186935 or vehicle control intravenously at a dose of 10 mg/kg once daily for 14 days. We observed a tumor growth inhibition (TGI) of 42.6% (*P* = 0.0006) on day 14 (Figure 4B). Treatment with this concentration of OTS186935 was well tolerated as indicated by the minimal relative body weight change (Figure 4C) without any detectable toxicity during the course of treatment. We then evaluated the *in vivo* growth suppressive effect of OTS186935 in a xenograft model of A549 human lung cancer cells. Intravenous administration of OTS186935 at 25 mg/kg once daily for 14 days yielded a TGI of 60.8% (*P* = 0.022) (Figure 4D) without significant body weight loss or toxicity (Figure 4E). Given this significant growth suppression, we further investigated the effect of OTS186935 inhibition treatment on the H3K9me3 status *in vivo*. We prepared nuclear extracts from excised xenograft tumors to elucidate whether OTS186935 could attenuate H3K9me3 levels in the mice treated with the compound. Western blot analysis showed attenuation of H3K9me3 levels in mice treated with OTS186935 compared to the control group, consistent with *in vitro* findings, demonstrating this compound’s ability to inhibit SUV39H2 enzymatic activity *in vivo* (Figure 4F). We also performed immunohistochemical (IHC) staining of tumor sections using a proliferation nuclear antigen Ki-67-specific antibody. IHC analysis of the excised tumors derived from the OTS186935-treated mice demonstrated a significantly lower percentage of Ki-67 positive cells compared to the control mice (*P* = 0.003) (Figure 4G), indicating suppression of A549 cancer cell proliferation.

### Enhanced anti-tumor effect of combination treatment *in vivo*

**Figure 5:**
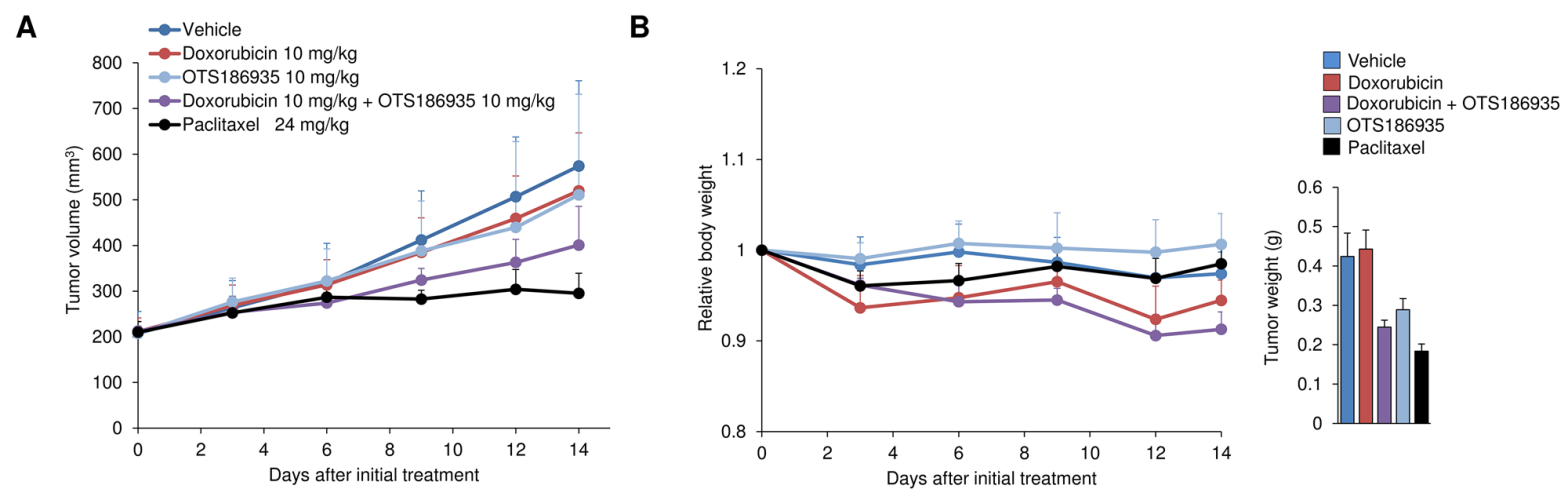
Evaluation of OTS186935 and DOX combination treatment in A549 xenograft mouse model **(A-B)** Additive growth suppressive effect of A549 cells *in vivo*. (A) Mice harboring A549 lung cancer cells (*n* = 6 per group) were intravenously treated with OTS186935, DOX, or the combination of both for 14 days. DOX was administered on day 2 and day 9 of treatment, and OTS186935 was administered once daily for 14 days. Combination treatment (OTS186935 and DOX) resulted in a TGI of 49% at day 14 compared to vehicle control. (B) Mean relative body weights + s.d. compared to mean body weight prior to administration of compounds (day 0), and mean tumor weight + s.d. on day 14 are shown.

After evaluating the *in vivo* efficacy of OTS186935 in two xenograft model systems, we investigated the effect of the compound in combination with DOX against A549 tumors *in vivo*. When the tumors reached a size of 200 mm^3^, DOX was administered intravenously at 10 mg/kg on day 2 and day 9 in combination with 10 mg/kg OTS186935 once daily for 14 days. At these respective doses we observed a TGI of 49% (*P* = 0.066, Student’s *t*-test) at the end of the study and combination treatment resulted in a slight reduction of body weight, however no overt toxicity was observed with this treatment regime (Figure 5A, 5B). These results demonstrate that at a relatively low concentration of each compound we can observe an additive growth suppressive effect *in vivo*.

## DISCUSSION

In the present study, we reported the development of novel SUV39H2 methyltransferase inhibitors, OTS193320 and OTS186935 (Figure 2A lower panel, 4A), with a common imidazo[1,2-*a*]pyridine scaffold (Figure 2A top panel), and characterized the biological importance of SUV39H2 inhibition in breast cancer cells. Many reports have provided mechanistic insight and have shown that deregulation of protein methyltransferases are intricately involved in human tumorigenesis [12–15, 21–27]. As such, SUV39H2-mediated methylation has an impact on tumorigenesis both through methylation of histone and non-histone substrates [12–15], and *SUV39H2* genetic alterations have been reported in many cancer types. Importantly, SUV39H2 expression was restricted to adult testis in normal tissues and was highly elevated in a variety of tumor types, thus making it an ideal target for anti-cancer drug development [12].

We showed that SUV39H2 was abundantly overexpressed in breast cancer cell lines using quantitative real-time PCR and western blot analysis, and siRNA-mediated SUV39H2 knockdown significantly decreased cancer cell viability *in vitro*. Incubation of two TNBC cell lines with OTS193320 attenuated H3K9me3 levels, in concordance with siRNA-mediated SUV39H2 knockdown. Given the importance of γ-H2AX production in chemoresistance, we addressed whether OTS193320 could affect γ-H2AX formation when it was used in combination with DOX. Combination of OTS193320 and DOX significantly attenuated cancer cell viability *in vitro*, compared to the treatment with a single agent, suggesting that OTS193320 may overcome the chemoresistance of cancer cells. Furthermore, the combination therapy reduced γ-H2AX levels in breast cancer cells, and further attenuated the formation of 53BP1/γ-H2AX foci, supporting that OTS193320 regulates the production of γ-H2AX in cancer cells. These results are in line with previous findings that attenuation of methylated H2AX enhances chemosensitivity of cancer cells [12]. We further demonstrate that OTS186935, an analog of OTS193320 with the common imidazopyridine scaffold, had a significant growth suppressive effect in xenograft mouse models of MDA-MB-231 and A549 cells, which showed high expression levels of SUV39H2. Intravenous administration of the OTS186935 inhibitor resulted in a TGI of 42.6% and 60.8% in the breast cancer and lung cancer mouse models, respectively (Figure 4B, 4D). This compound displayed a potent anti-tumor effect associated with reduction of H3K9me3 levels and Ki-67 nuclear positivity, and was well tolerated during the treatment (Figure 4F, 4G).

TNBCs are characterized by the absence of the estrogen and progesterone hormone receptors as well as HER2 overexpression, and their treatment remains a challenge due to the lack of defined targeted therapies [28, 29]. These tumors exhibit molecular heterogeneity and distinct gene expression signatures, which further indicate the need for appropriately tailored patient-specific targeted therapy [30, 31]. The current standard of care for TNBCs is chemotherapy-based regimes. In the neoadjuvant setting, systemic chemotherapeutic agents including anthracyclines, taxanes and cyclophosphamide, have been used to increase an opportunity for breast conserving surgery [32–35]. In addition, novel promising agents and different drug combinations are currently being explored to fight against the aggressive nature of the disease. On account of γ-H2AX production conferring poor prognosis in different types of cancer including TNBC and NSCLC, and our *in vivo* efficacy of SUV39H2 inhibition in two model systems, SUV39H2 may possess therapeutic potential in a wide range of human cancers.

In conclusion, we have characterized the biological effects of SUV39H2 inhibition by OTS193320 *in vitro* on breast cancer cell viability and γ-H2AX regulation, and further demonstrated the *in vivo* growth suppressive efficacy of OTS186935 in breast and lung cancer models without any visible toxicity. Our data have shown that SUV39H2 inhibition plays important regulatory roles in γ-H2AX production in cancer cells by these two small-molecular compounds. These results indicate that SUV39H2 inhibition overcomes chemoresistance of cancer cells and indicate that targeting SUV39H2 may avail as a novel treatment strategy in various cancer types.

## MATERIALS AND METHODS

### Cancer cell lines

MDA-MB-231, BT-549, BT-20, HCC1937, MCF-7, T-47D, ZR-75-1, SK-BR-3 cell lines were obtained from American Type Culture Collection (ATCC) (Manassas, VA, USA). All cell lines were grown in monolayers in appropriate media supplemented with 10% fetal bovine serum and 1% antibiotic/antimycotic solution (Sigma-Aldrich): RPMI 1640 Medium for BT-549, T-47D, HCC1937 and ZR-75-1 cells; Minimum Essential Medium for MCF-7 and BT-20 cells; McCoy’s 5A Medium for SK-BR-3 cells; Leibovitz’s L-15 Medium for MDA-MB-231 cells. All cells were maintained at 37°C in humid air with 5% CO_2_ condition (BT-549, T-47D, HCC1937, ZR-75-1, MCF-7, BT-20 and SK-BR-3), or without CO_2_ (MDA-MB-231). All cell lines were authenticated by STR analysis.

### Antibodies

The following primary antibodies were used for western blot (WB) and immunocytochemical (ICC) analysis: Anti-Histone H3 (ab1791); Abcam; dilution used in WB: 1:20000, Anti-Histone H3 (tri methyl K9) (ab8898); Abcam; dilution used in WB: 1:10000; dilution used for ICC: 1:500, Anti-α-tubulin (DM1A) (mouse #CP06); Calbiochem; dilution used for ICC: 1:100, Anti-Caspase-3 (8G10) (rabbit #9665); Cell Signaling Technology; dilution used in WB: 1:1000, Anti-Caspase-8 (1C12) (mouse #9746); Cell Signaling Technology; dilution used in WB: 1:1000, Anti-Caspase-9 (C9) (mouse #9508); Cell Signaling Technology; dilution used in WB: 1:1000, Anti-β-Actin (AC-15) (mouse #A5441); Sigma-Aldrich; dilution used in WB: 1:5000, Anti-phospho-Histone H2A.X (Ser139) (mouse #05-636); Millipore; dilution used in WB: 1:2000; dilution used for ICC: 1:250, Anti-Histone H2A.X (rabbit #2595); Cell Signaling Technology; dilution used in WB: 1:1000, Anti-53BP1 (rabbit #4937); Cell Signaling Technology; dilution used for ICC: 1:100. Anti-SUV39H2 monoclonal antibody was generated by immunizing BALB/cCrSlc mice with recombinant SUV39H2 into abdominal cavity, followed by six additional injections. Spleen cells from the mice were used to generate hybridomas by fusion with Sp2/O-Ag14 myeloma cells with polyethylene glycol 1500. Then, the hybridoma clone (11D10D9) which produces antibodies reacting with SUV39H2 on WB, dilution 1:1000 was isolated.

### Quantitative real-time PCR

Specific primers for human *GAPDH* (housekeeping gene) and *SUV39H2* were designed (detailed primer sequences in Supplementary Table 1). PCR reactions were performed using the ViiA 7 Real-Time PCR System (Thermo Fisher Scientific) following the manufacturer’s protocol. Messenger RNA levels were normalized to *GAPDH* expression level.

### Western Blotting

Nuclear extracts were prepared using the Nuclear Extract kit (Active Motif) to examine protein levels of SUV39H2 and H3K9me3; whole cell lysates were prepared using CelLytic^™^ M mammalian cell lysis reagent (Sigma-Aldrich, St. Louis, MO, USA) supplemented with a complete protease inhibitor cocktail (Roche Applied Science) and a phosphatase inhibitor cocktail (Roche Applied Science) to examine levels of apoptotic markers. Purified core histones were obtained using the Histone Purification Mini Kit (Active Motif). Nuclear extracts, whole cell lysates and purified histone extracts were separated by SDS-PAGE and blotted to nitrocellulose membrane. Nitrocellulose membrane was first incubated with each primary antibody as described in the *"Antibodies"* section and then protein bands were detected by incubating the secondary antibody, horseradish peroxidase (HRP)-conjugated antibodies and visualized with Amersham^™^ ECL^™^ Western Blotting Detection Reagents (GE Healthcare, GE Healthcare, Little Chalfont, UK) or Amersham^™^ ECL^™^ Prime Western Blotting Detection Reagent (GE Healthcare, GE Healthcare, Little Chalfont, UK). Raw data is provided in Supplementary Materials.

### Kaplan–Meier survival analysis

Kaplan–Meier survival analysis of breast cancer patients was performed using SUV39H2 expression levels in cancer tissues utilizing an online survival analysis tool (Kaplan–Meier Plotter) [20]; www.kmplot.com. The expression range of the *SUV39H2* probe (1554572_a_at) was 28 to 4984 in the breast cancer data set, and the cutoff level used in the analysis was 324 with patients being split by the median expression value to calculate the relapse-free survival (RFS) rate (*n* = 1764).

### siRNA transfection and cell viability assays

MISSION_ siRNA oligonucleotide duplexes were purchased from Sigma–Aldrich for targeting the human SUV39H2 transcripts (SASI_Hs01_00101226 and SASI_Hs02_00357085). siNegative control (siNC), which consists of three different oligonucleotide duplexes, were used as control siRNAs (Cosmo Bio, Tokyo, Japan). The siRNA sequences are described in Supplementary Table 2. For knockdown studies, breast cancer cells were plated overnight in 100-mm culture dishes and were transfected with siRNA duplexes (final concentration of 50 nM) using Lipofectamine RNAimax (Thermo Fisher Scientific) for 72 hours (3 days) at a confluence of ∼ 50%. For cell viability assays, breast cancer cells were plated overnight in quadruples in 24-well plates (2 × 10^4^ cells/well) and were transfected with siRNA duplexes (final concentration of 50 nM) using Lipofectamine RNAimax (Thermo Fisher Scientific) for 5 days with re-transfection on day 4. The number of viable cells was assessed using the Cell Counting Kit-8 system (Dojindo, Kumamoto, Japan) on day 5 [12]. For apoptosis analysis, breast cancer cells were plated in 100-mm culture dishes overnight in triplicates and the following day exposed to OTS193320. After incubation with the compound for 48 hours, the cells were collected and apoptosis was measured using the Annexin V Apoptosis Detection Kit (eBioscience) according to the manufacturer’s protocol.

### Development of small-molecular compounds

Compounds OTS193320 and OTS186935 were synthesized according to the procedures as described elsewhere (Patent WO2017058503), by contract with Albany Molecular Research, Inc.

### IC_50_ determination

Cells were seeded in 96-well plates at a density of 4 × 10^3^ and were allowed to adhere for 24 hours. On the following day, the medium was exchanged and cells were exposed to the compound for 72 hours. The Cell Counting Kit-8 system (Dojindo, Kumamoto, Japan) was used to examine cell viability and the plates were read in a microplate reader at 450 nm. IC_50_ values were calculated using GraphPad Prism software.

### *In vitro* methyltransferase assay

The compounds OTS193320 or OTS186935 were serially diluted (10 doses) and mixed with biotin conjugated histone H3 peptide (1-21) (final conc. 350 nM) (Millipore: 12-403) and adenosyl-L-Methionine, S-[Methyl-^3^H]- (final conc. 100 nM) (Perkin Elmer: NET155H001MC) in the assay buffer (20 mM Tris pH 8.0, 10 mM MgCl_2_, 50 mM NaCl, 10 mM DTT, 0.03% Tween-80). N-terminal GST fused SUV39H2 (final conc. 20 nM) (in-house) was added to start reaction and incubated at room temperature for 3 hours. The reaction was stopped by adding 2.5 mg/mL of Streptavidin SPA beads in bead buffer (20 mM Tris pH 8.0, 500 mM MgCl_2_, 50 mM NaCl, 10 mM DTT, 0.03 % Tween-80). The radioactive signal was measured with Trilux-Microbeta counter (Perkin Elmer). IC_50_ was calculated with XLfit.

### Immunocytochemistry

Cells were fixed in 4% paraformaldehyde in 0.1 M phosphate buffer at 4°C for 1 h, then permeabilized in 0.1% Triton X-100 (Sigma-Aldrich) for 3 min at room temperature, and blocked with 3% BSA for 1 h at room temperature. Fixed cells were incubated with primary antibodies overnight at 4°C, followed by incubation with Alexa Fluor-conjugated secondary antibodies (Life Technologies, Carlsbad, CA, USA) [12], and observed using Leica confocal microscopy (SP5 tandem Scanner Spectral 2-Photon Confocal).

### *In vivo* xenograft study

The animal experiments were performed at OncoTherapy Science, Inc. in accordance with their Institutional Guidelines for the Care and Use of Laboratory Animals. MDA-MB-231 cells (1 × 10^7^ cells) were injected subcutaneously in the left flank of female NOD.CB17-Prkdcscid/J mice (Charles River Laboratory). A549 cells (1 × 10^7^ cells) were injected subcutaneously in the left flank of female BALB/cAJcl-*nu/nu* mice (CLEA Japan, Inc.). When MDA-MB-231 and A549 xenografts reached approximately 150 to 250 mm^3^, animals were randomized into groups. Number of mice in a group was 6 for the study with MDA-MB-231 and A549 in combination with DOX, and 3 for the study with A549 and single treatment of OTS186935. For intravenous administration, OTS186935 and doxorubicin hydrochloride (Kyowa Hakko Kirin Co. Ltd.) was formulated in a 5% glucose solution and 0.9% sodium chloride injection (Otsuka Pharmaceutical Co., Ltd.) and injected via tail vein. An administration volume of 10 mL/kg of body weight was used. The doses administered were indicated in the main text and figures. Tumor sizes were measured every other day or twice weekly by using a caliper. The results were converted to tumor volumes (mm^3^) by the formula length × width^2^ × 1/2. Tumor growth inhibition (TGI) was determined by the formula {1 – (*T* – *T*_0_) / (*C* – *C*_0_)} · 100, where *T* and *T*_0_ are the mean tumor volumes on day 14 and day 0, respectively, for treatment groups, and *C* and *C*_0_ are those for the vehicle control group. Tumor mass was dissected from mice on day 14 and tumor weight was determined and then fixed with 4% paraformaldehyde.

### Immunohistochemical staining

After deparaffinization and rehydration, tissue sections were treated with antigen retrieval solution (DAKO, S2367) in a steamer for 20 minutes. Anti-Ki67 antibody (Thermo Scientific Labvision, Cat#RM-9106-s, rabbit monoclonal antibody, Clone: SP6, dilution 1:300) was applied on tissue sections for one hour incubation at room temperature in a humidity chamber. Following TBS wash, the antigen-antibody binding was detected with Envision+ system (DAKO, K4003) and DAB+ chromogen (DAKO, K3468). Tissue sections were briefly immersed in hematoxylin for counterstaining and were covered with cover glasses.

## SUPPLEMENTARY MATERIALS FIGURE AND TABLES


