# Supplementary material for: Development of novel SUV39H2 inhibitors that exhibit growth suppressive effects in mouse xenograft models and regulate the phosphorylation of H2AX
Source: Oncotarget. 2018 Aug 7;9(61):31820–31. doi: 10.18632/oncotarget.25806 (PMC6112750; doi:10.18632/oncotarget.25806)
Supplement: Supplementary file 1 [file oncotarget-09-31820-s001.pdf]

Development of novel SUV39H2 inhibitors that exhibit growth suppressive effects in mouse xenograft models and regulate the phosphorylation of H2AX

SUPPLEMENTARY MATERIALS

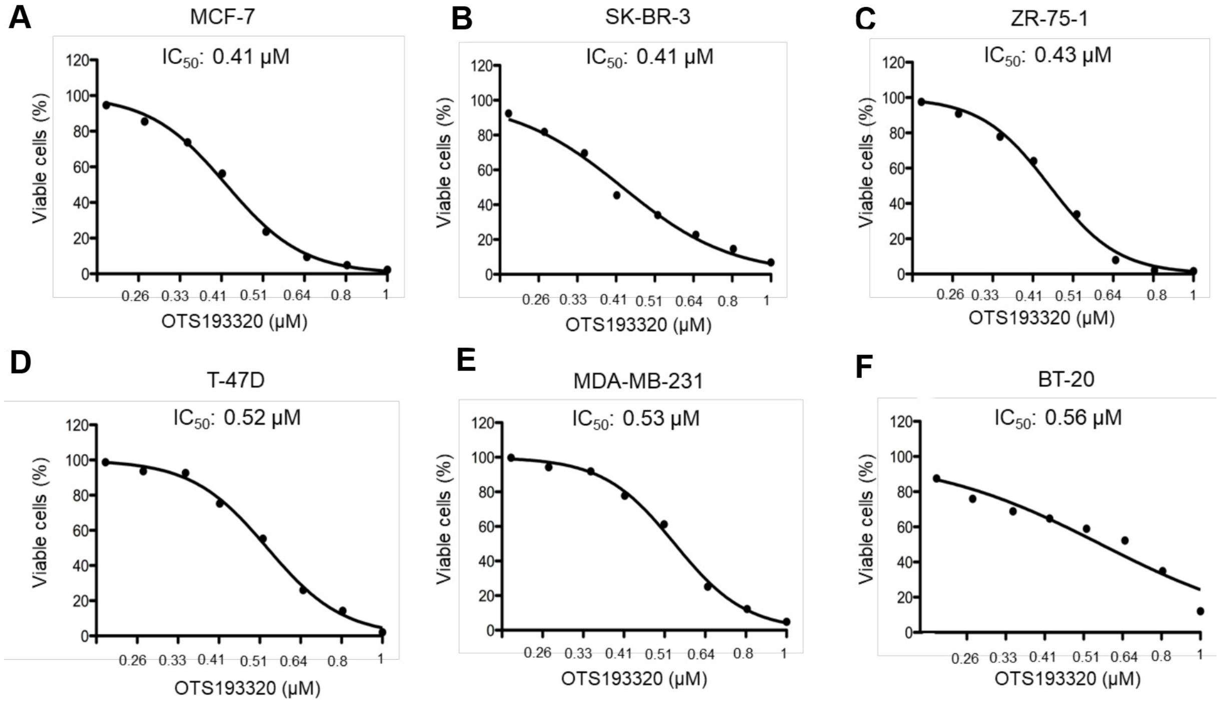

Supplementary Figure 1: Growth suppressive effect of OTS193320 in breast cancer cell lines. *In vitro* anti-proliferative effect of OTS193320 in six breast cancer cell lines, **(A)** MCF-7, **(B)** SK-BR-3, **(C)** ZR-75-1, **(D)** T-47D, **(E)** MDA-MB-231, **(F)** BT-20. Graphs indicate IC_{50} values after exposure to OTS193320 for 72 hours.

Supplementary Table 1: Primer sequences for quantitative real-time PCR

Gene name	Primer sequence
GAPDH (housekeeping gene)-f	5’ GCAAATTCCATGGCACCGTC 3’
GAPDH (housekeeping gene)-r	5’ TCGCCCCACTTGATTTTGG 3’
SUV39H2-f	5’ TGGGGTGTAAAGACCCTTGTG 3’
SUV39H2-r	5’ ATTCCCTTGTTGTCATAGAAC 3’

Supplementary Table 2: siRNA sequences

siRNA name		Sequence
	Target#1	Sense: 5’ AUCCGCGCGAUAGUACGUA 3’
siNegative control		Antisense: 5’ UACGUACUAUCGCGCGGAU 3’
(Cocktail)	Target#2	Sense: 5’ UUACGCGUAGCGUAAUACG 3’
		Antisense: 5’ CGUAUUACGCUACGCGUAA 3’
	Target#3	Sense: 5’ UAUUCGCGCGUAUAGCGGU 3’
		Antisense: 5’ ACCGCUAUACGCGCGAAUA 3’
siSUV39H2#1		Sense: 5’ CUUUGGUUGUUCAUGCACA 3’
		Antisense: 5’ UGUGCAUGAACAACCAAAG 3’
siSUV39H2#2		Sense: 5’ GUGAAGAAGGCUAAACAAA 3’
		Antisense: 5’ UUUGUUUAGCCUUCUUCAC 3’

RAW DATA

1B

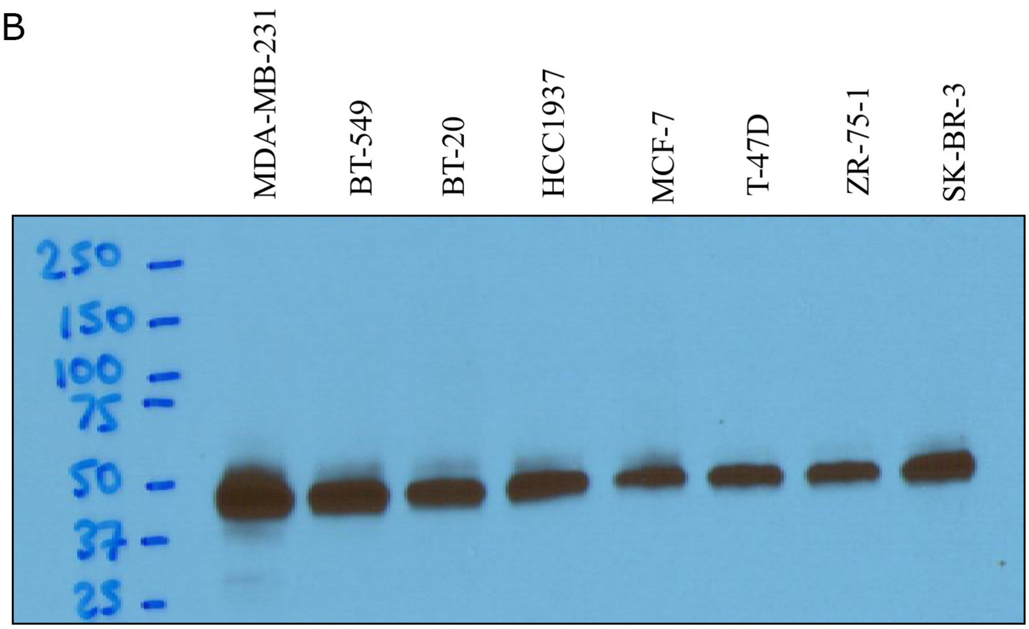

IB:SUV39H2

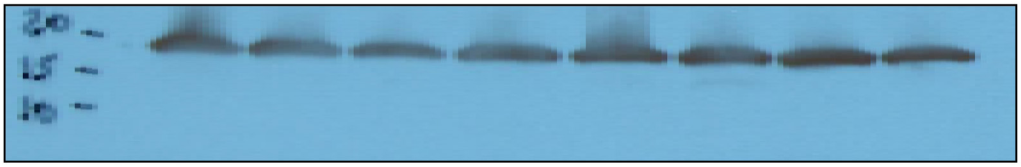

IB:Histone H3

1D

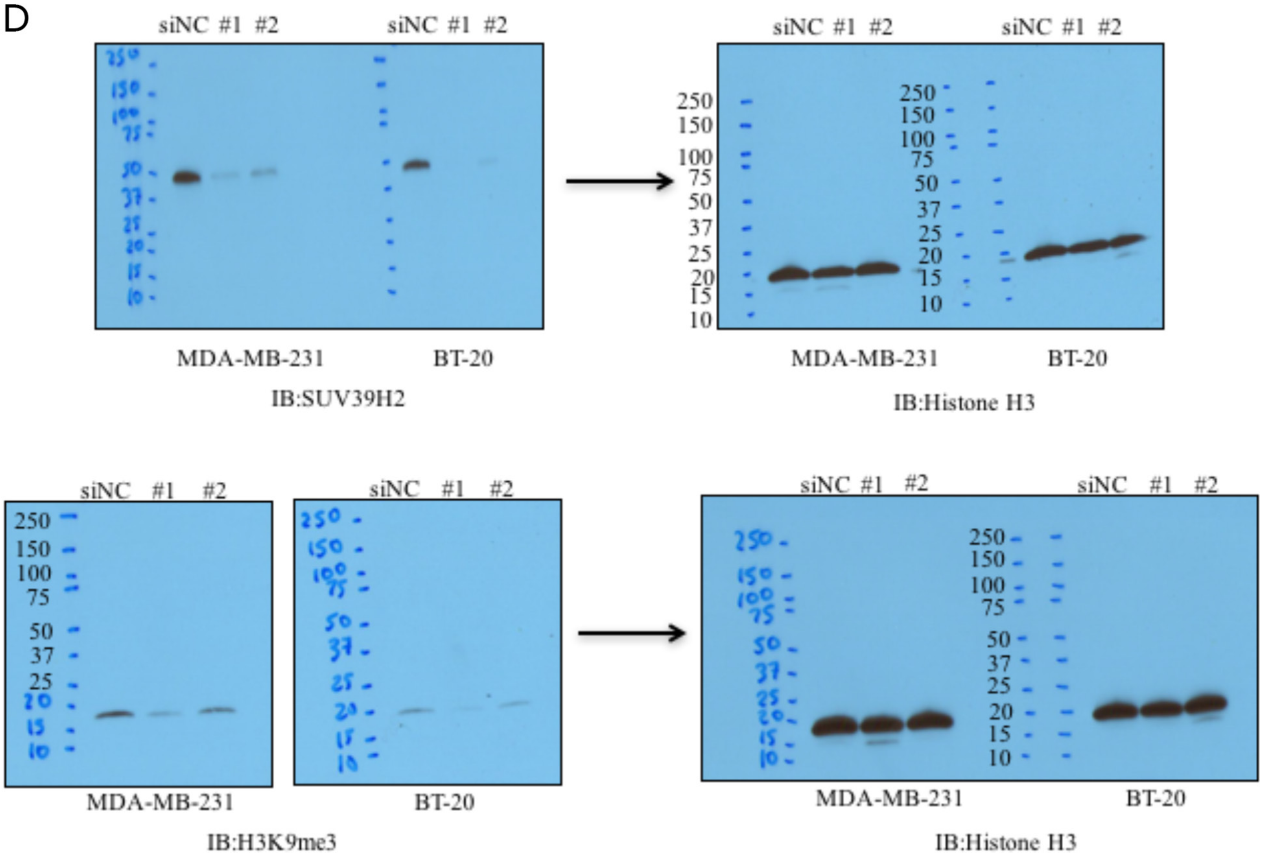
siNC #1 #2
siNC #1 #2
MDA-MB-231
BT-20
IB:SUV39H2
siNC #1 #2
siNC #1 #2
250
150
100
75
50
37
25
20
15
10
MDA-MB-231
BT-20
IB:Histone H3
siNC #1 #2
siNC #1 #2
MDA-MB-231
BT-20
IB:H3K9me3
siNC #1 #2
siNC #1 #2
MDA-MB-231
BT-20
IB:Histone H3

2B

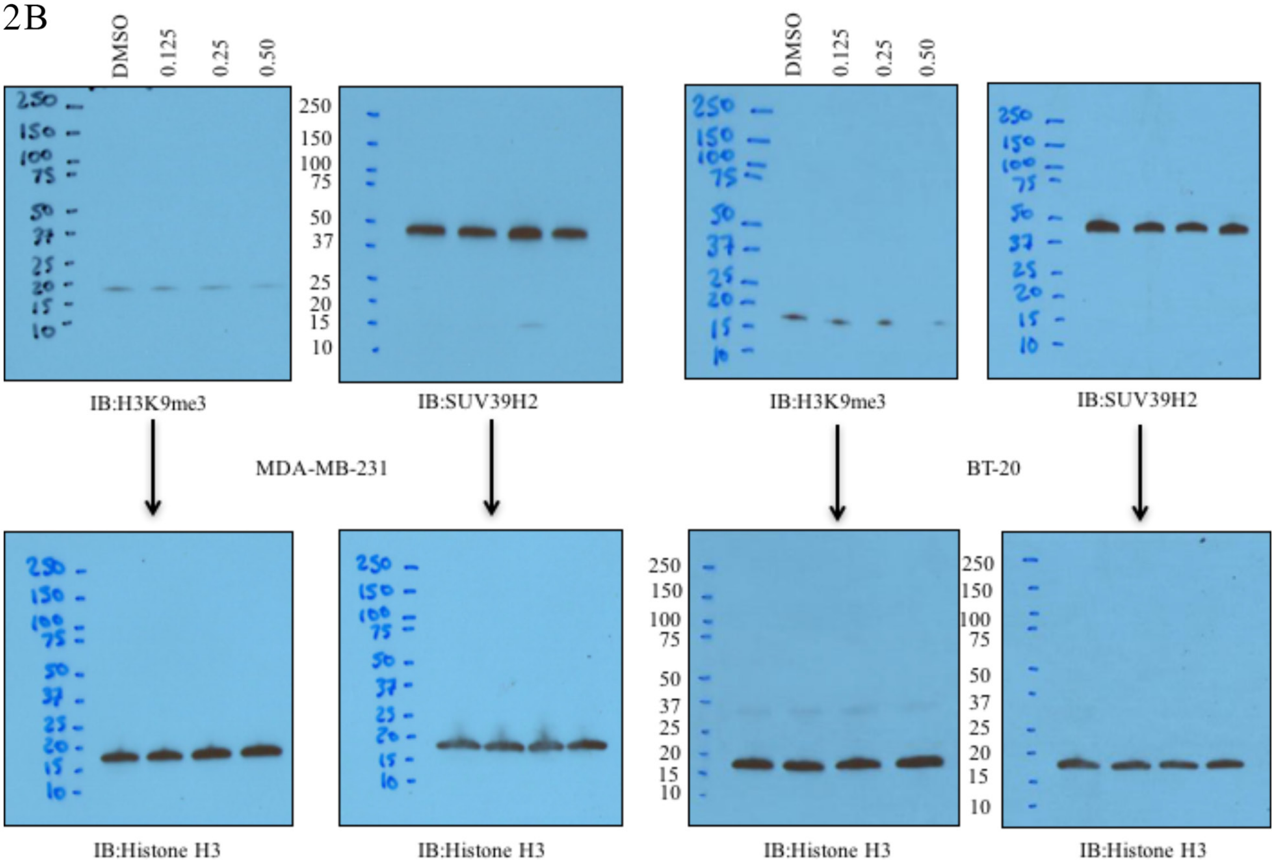
DMSO
0.125
0.25
0.50
IB:H3K9me3
250
150
100
75
50
37
25
20
15
10
IB:SUV39H2
DMSO
0.125
0.25
0.50
IB:H3K9me3
IB:SUV39H2
MDA-MB-231
BT-20
IB:Histone H3
IB:Histone H3
IB:Histone H3
IB:Histone H3

2E

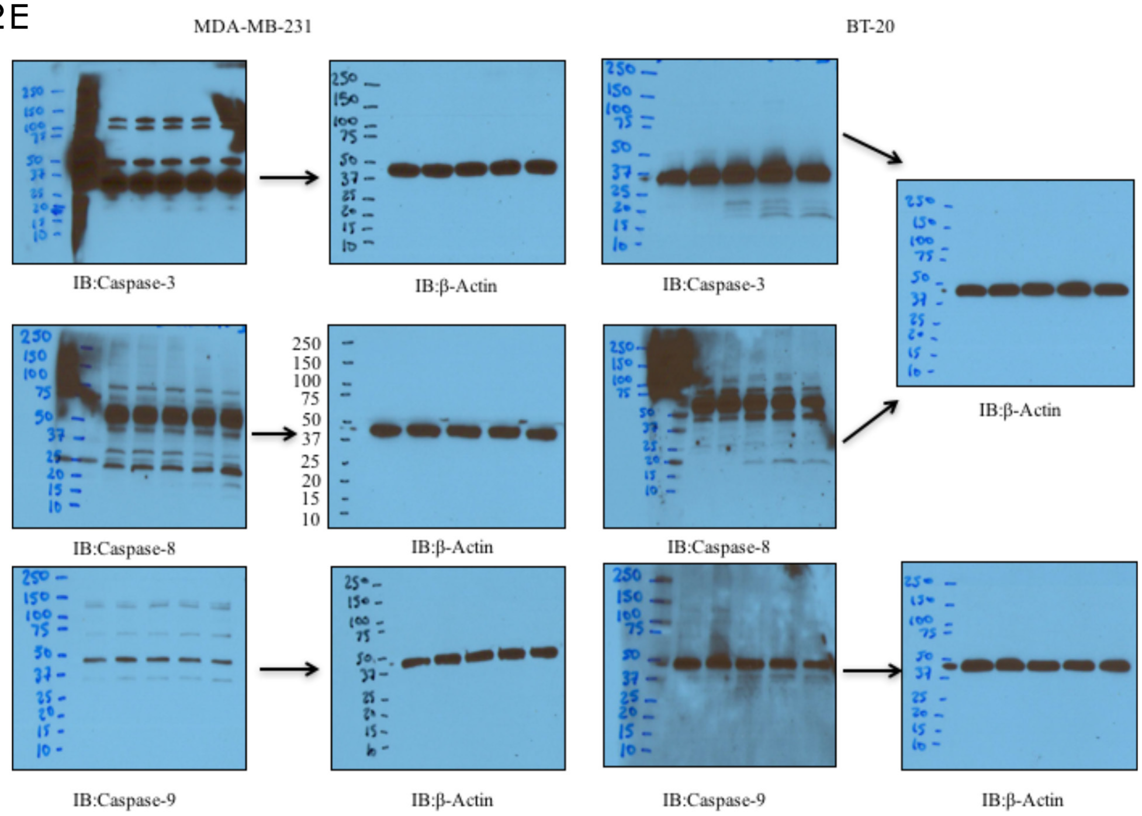
MDA-MB-231
BT-20
IB:Caspase-3
IB:β-Actin
IB:Caspase-3
IB:β-Actin
IB:Caspase-8
IB:β-Actin
IB:Caspase-8
IB:Caspase-9
IB:β-Actin
IB:Caspase-9
IB:β-Actin
250
150
100
75
50
37
25
20
15
10

3B

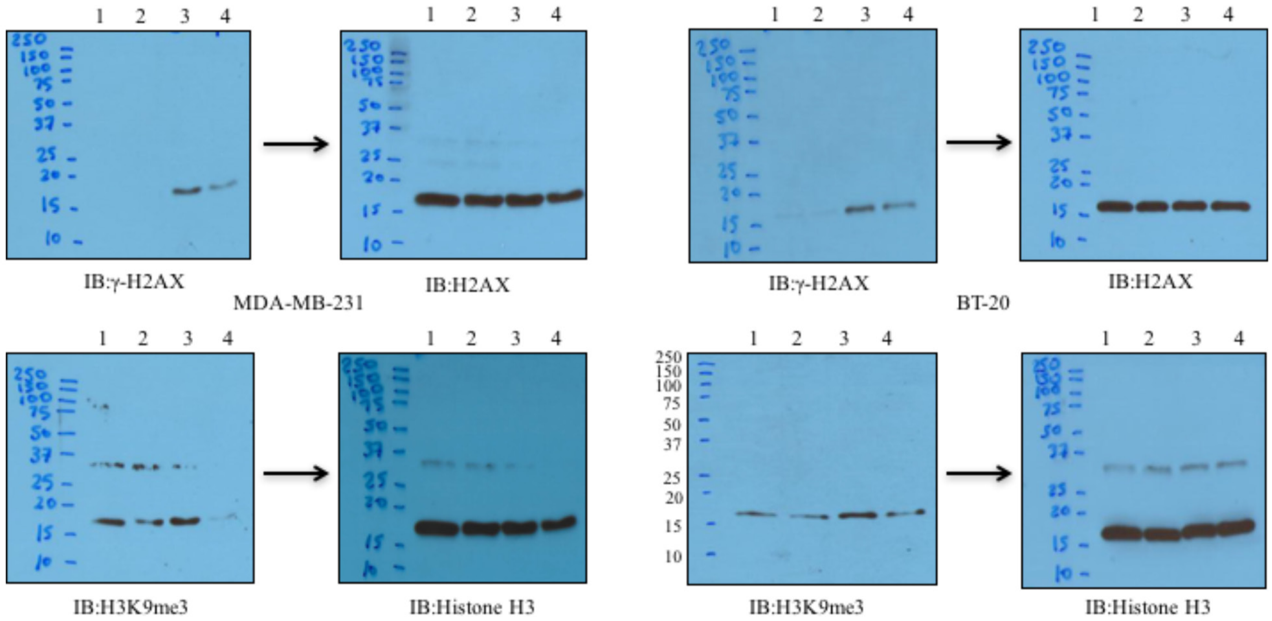
1 2 3 4
IB:γ-H2AX
IB:H2AX
MDA-MB-231
IB:γ-H2AX
IB:H2AX
BT-20
IB:H3K9me3
IB:Histone H3
IB:H3K9me3
IB:Histone H3
250
150
100
75
50
37
25
20
15
10

4F

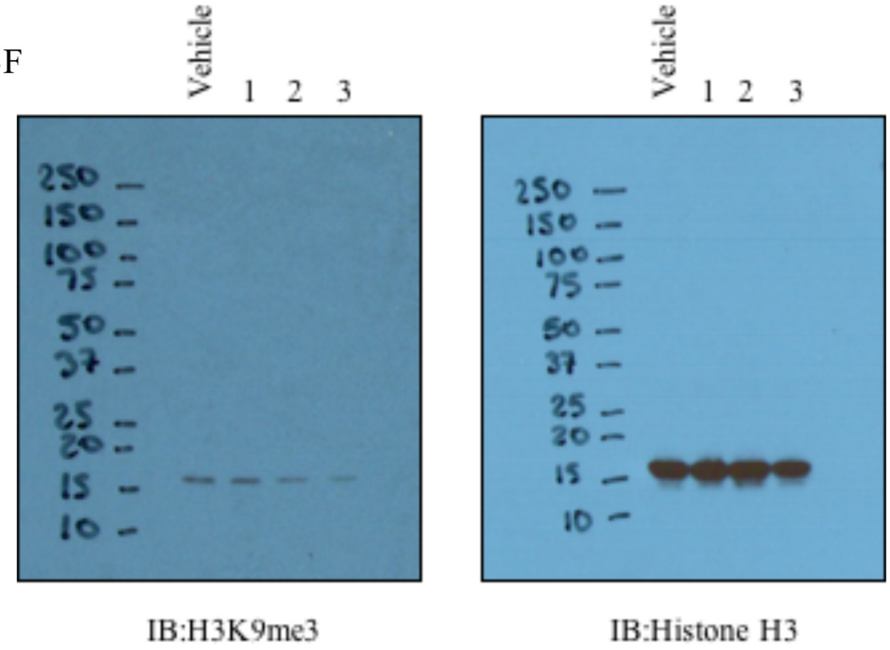
Vehicle
1
2
3
250
150
100
75
50
37
25
20
15
10
IB:H3K9me3
Vehicle
1
2
3
250
150
100
75
50
37
25
20
15
10
IB:Histone H3
